# “An Environment Built to Include Rather than Exclude Me”: Creating Inclusive Environments for Human Well-Being

**DOI:** 10.3390/ijerph120911146

**Published:** 2015-09-08

**Authors:** Natasha A. Layton, Emily J. Steel

**Affiliations:** 1 School of Health and Social Development, Deakin University, Burwood, Victoria 3125, Australia; 2 School of Human Services and Social Work, Griffith University, Meadowbrook 4131, Australia; E-Mail: emjsteel@gmail.com; 3 School of Health and Rehabilitation Sciences, the University of Queensland, St Lucia 4072, Australia

**Keywords:** occupational therapy, inclusion, disability, ICF, environmental factors, health policy, accessibility, usability

## Abstract

Contemporary discourses which challenge the notion of health as the “absence of disease” are prompting changes in health policy and practice. People with disability have been influential in progressing our understanding of the impact of contextual factors in individual and population health, highlighting the impact of environmental factors on functioning and inclusion. The World Health Organization’s (WHO) more holistic definition of health as “wellbeing” is now applied in frameworks and legislation, and has long been understood in occupational therapy theory. In practice, however, occupational therapists and other professionals often address only local and individual environmental factors to promote wellbeing, within systems and societies that limit equity in population health and restrict inclusion in communities. This paper presents an in-depth analysis of the supports and accommodations identified by a cohort of individuals (n-100) living with disability. A range of environmental facilitators and barriers were identified in peoples’ experience of “inclusive community environs” and found to influence inclusion and wellbeing. The roles and responsibilities of individuals, professionals, and society to enact change in environments are discussed in light of these findings. Recommendations include a focus on the subjective experience of environments, and application of theory from human rights and inclusive economics to address the multiple dimensions and levels of environments in working towards inclusion and wellbeing.

## 1. Introduction

If I lived in a society where being in a wheelchair was no more remarkable than wearing glasses, and if the community was completely accepting and accessible, my disability would be an inconvenience and not much more than that. It is society which handicaps me, far more seriously and completely than the fact that I have Spina Bifida.

*[1] (p. 12)*

This statement by an Australian living with disability challenges the assumption that having an impairment (in the writer’s words, “disability”) is a major limitation. Instead, social and physical environments are identified as barriers to wellbeing, and the cause of disability; an idea encapsulated by the social model of disability [2]. Disability theorists also tell us that living with variations in or, to use the International Classification of Functioning, Disability and Health’s (ICF) terminology, impairment of body structures and functions, is not necessarily a tragic or negative circumstance, but instead can be an interesting and even satisfying experience of corporeal difference [3,4]. The social model of disability deconstructs and debunks traditional biomedical discourses that draw a causal relationship between impairment and disability without regard for the environment [5,6]. However, neither biomedical nor social models acknowledge a mutual relationship between the environment and health, instead asserting either no role at all for the environment or a causal role in disability.

Turning to consider what theories of environment have to say about disability and inclusion, a range of literatures contain relevant, if partial, perspectives. Ecological models of health explain how the environment influences human behavior, but also how humans shape their environments around their activities. Public health and community development literature acknowledges that people participate in healthy activities when they live in environments that afford them access to cultural, economic, and social occupations [7]. Thus, the environments in which daily activities are carried out are directly and critically related to health and wellbeing [8,9]. Approaches including Community Based Rehabilitation (CBR) and the Sustainable Livelihoods Framework are used to address inequalities and build social capital, by identifying and utilizing resources in the environment and processes that influence wellbeing [10,11]. These bodies of work identify that environmental factors can either facilitate or restrict participation in society and thus affect the health of populations.

Going one step further to enact a biopsychosocial model of health is the WHO’s ICF [12]. The ICF recognizes the central role played by environmental factors and places the focus of intervention upon individuals as well as the environment in which the individual lives. It normalizes environmental barriers and facilitators as an experience for people with and without impairment. The ICF also offers the qualifiers of environmental factors as barriers or facilitators, providing a lens through which to view the transaction of the environment and the individual. This recognizes that the way in which certain environmental factors are experienced by individuals makes them into either facilitators or barriers:

Environmental factors are to be coded from the perspective of the person whose situation is being described. For example, kerb cuts without textured paving may be coded as a facilitator for a wheelchair user but as a barrier for a blind person.

*[12] (p. 171)*

Importantly, the ICF encompasses tangible and intangible elements of environments within its Chapters: Ch. 1 Products and Technology; Ch. 2 Natural environment and human-made changes to environment; Ch. 3 Support and Relationships; Ch. 4 Attitudes; Ch. 5 Services, systems and policies [12]. 

Developed contemporaneously with the ICF, the Disability Creation Process [13] also articulates dynamic interactions between environmental factors (social and physical) and social participation at an individual level, and operationalizes participation more clearly that the ICF [14]. Despite this, the ICF is currently considered the “global standard for describing and characterizing aspects of disability” [9] (p. 169), and is used in this paper because of its broad adoption across disciplines and in international disability rights discourses [15].

Drawing these threads together, disability is no longer seen as the result of an individual’s impairment, as approaches to disability have broadened in focus from individuals and their immediate physical environment, to consider additional dimensions of the environment (e.g., social, institutional, virtual) and broader societal structures [16]. 

The term disablement can be used to emphasize the potentially disabling impact of environmental barriers for people with impairment [17]. The locus of disablement occurs in the space between individuals’ capabilities, the tasks they aspire to, and the environments in which they exist. Disability can therefore be defined as impairment of body structure or function combined with subjective experience of disablement brought about by environmental barriers. Should environmental barriers exist, the effects of impairment are magnified and the person experiences disablement. As Whiteneck *et al.* note,

People with disability experience more frequent and/or more problematic barriers than people without disabilities and … the nature and severity of the disability relates to the frequency and magnitude of the barriers encountered.

*[18] (p. 1329)*

There is however a lack of “empirical research as to the fundamental question of how intrinsic features of an individual interact with features of the social environment to produce disablement” [19] (p. 1174) [20]. 

This paper firstly seeks to fill this evidence gap by asking “how do people’s experiences of community environments inform our understanding of the relationship between environment and inclusion?”

A subsequent question then needs to be answered: if people with disability cannot access their environments and therefore cannot engage in the daily occupations, is there a shared (societal) responsibility for the structural restrictions on one’s health that cannot be addressed by individual agency alone? This question raises the notion of the social contract between people with disability and society, expressed in legal and moral terms by human rights conventions that set the context for public policy, such as the Convention on the Rights of Persons with Disabilities (CRPD) [21]. Current evidence suggests governments in Australia are not yet fulfilling this social contract and achieving the stated policy goal of equal outcomes for people with disability through the provision of necessary mediators and supports [22].

Our society’s places and practices of exercising, sharing or wielding power systematically exclude people with disabilities. Indeed, we suspect that people with disabilities have long been on the margins of Australian political life—although there has been a conspicuous lack of interest in and research on this topic.

[22] (p. 142)

The notion of a shared responsibility for health and inclusion through environmental intervention provides the impetus for this paper, which aims to inform change and improvement in the way individuals, professionals, and society enact change in environments for inclusion and wellbeing. 

As illustrated by the introductory quote, the lived experience, and perspective, of the person with disability provides a critically important lens through which the fit between a person, their occupations and environment is understood. People with disability argue for subjectivity in the assessment of accessibility, and consideration of environments based on their individual experiences, rather than reliance on objective measures and assumptions of human abilities [23]. The inclusive methodology of the study embraces this subjective stance. Analysis for the purposes of this paper specifically focusses on quantitative and qualitative data related to the environment, to answer the research question, “how do people’s experiences of community environments inform our understanding of the relationship between environment and inclusion?”, and “how should professionals use this understanding of environment to promote inclusion?”

The findings demonstrate the multi-dimensional and subjective nature of the environment, its interactions with individuals, and significant relationship to inclusion and wellbeing. The paper concludes by discussing how theory can be applied in collective action for the creation of inclusive environments.

## 2. Experimental Section 

### 2.1. Study Aims and Context

In 2008 in the Australian state of Victoria, an alliance of assistive technology stakeholders [24], including people with disability, obtained philanthropic funding to commission two studies (The Equipment Study and The Economic Study) to answer the research question “What difference does Assistive Technology (AT) make to life for people with a disability?” The studies were commissioned as an act of social change, with the directive to ‘inform and effect real change in the way aids and equipment policies, programs, and supports are delivered’ [25] (p. 32). Reports were published in the grey literature in 2010 as The Equipping Inclusion Studies [25]. 

The Equipment Study focused on the experience of adults using Assistive Technology (AT) and the impact of this in their lives, and formed the basis of the first author’s PhD thesis [26]. The Equipment Study was a mixed method inquiry into life for 100 adults and elders living with disability in Victoria, Australia, in 2009–2010. Utilizing an inclusive research methodology [27], people with disability directed the research questions and were actively involved in data analysis via an advisory panel, as well as the pursuit of an improved policy agenda for Victoria based on the study findings. This paper presents a detailed analysis of a subset of data concerning the impact of environments, exploring community inclusion through the lens of a biopsychosocial model of health and the environment.

### 2.2. Methods 

Ethical approval was sought and gained from Deakin University Human Research Ethics Committee (Ethics Approval Number EC 5-2009}, with management of all data, storage, confidentiality, and reporting of results conducted in line with Australia’s National Ethics Guidelines. Ethical clearance was granted for use and reuse of data; and the commissioning Alliance directed researchers to disseminate the findings widely.

#### 2.2.1. Recruitment

A maximum variation sample was sought to elicit a wide range of data concerning life with disability and the nature of supports. Participation was invited across all impairment types, living situations, genders, and adult age groups, and $A20 gift cards were offered in recognition of participation. Electronic and paper flyers and surveys were sent to specialist disability organizations, AT user groups, Disabled People’s Organizations (DPOs) and rehabilitation facilities within Victoria, Australia. Snowball sampling also occurred, and recruitment efforts ceased when n-100 was reached over a three month period when funds were expended. 

#### 2.2.2. Participants

Adults who used AT, environmental modifications, and/or personal support were invited to participate. Robust methods of access support were used to include participants who required alternative access (for example switching or screen-reading software) or supported methods (such as proxy reporting or scribing for survey completion). Participants under the age of 18 were excluded as the range of life outcome areas on the survey tool did not encompass specific domains for children and adolescents. 

The survey allowed optional completion of all questions, and of the 100 respondents, 79 provided demographic data. Respondents identified having between one and twelve impairments or long-term health conditions, and reported nearly 60 separate diagnoses, the majority relating to physical impairment (59%), followed by multiple (14%), sensory (14%), psychological (6%) and neurological (5%) impairments. Nine percent of the sample identified cognitive impairments such as acquired brain injury or as secondary effects of other conditions such as the late effects of polio. There were more female (59%) than male (41%) respondents, grouped in age from 18 to 24 (7%), 25 to 44 (26%), 45 to 64 (50%), and over 65 (16%). Respondents’ living situations differed, with the majority living in private dwellings (92%). Of these, 65% reported living with a spouse or partner, and 27% with family members. Two percent of respondents lived in a supported group home, and a further 2% lived in a larger congregate care residence. Seventy four percent were unemployed, with 21% engaged in volunteer work and 4% interested in working or volunteering but reporting they are currently unable to due to lack of suitable supports and accommodations.

#### 2.2.3. Data Collection

Data was collected via a survey (accessible online format or paper format) comprising 97 questions including: (1) a series of ratings (difficulty, satisfaction, participation and time-use) and open fields to capture the impact of current and desired supports (AT, environmental interventions and personal care) across a range of life areas; (2) the Assessment of Quality of Life (AQoL) instrument [28], a standardized health-related quality of life measure, and; (3) demographic information. The survey was designed to identify the widest range of supports or accommodations and elicit general themes, so utilized a largely open-ended question set. Table 1 illustrates the support categories captured. All responses were entered online (by participants or their proxies, or by researchers, if the participants elected to provide survey question answers face-to-face or over the phone) and saved in an excel spreadsheet. This paper primarily draws on responses from Section 1).

The survey tool was piloted (n-4) for accessibility and content validity by community-dwelling adults with impairments including sensory, motor and cognition, of both genders, from younger and older age groups. The only proposed change concerned amending the standardized quality of life instrument because it failed to capture life for wheelchair users, as the mobility question concerned “walking”. Permission was granted from the AQoL authors to use the term “mobility” in lieu of walking, and no other amendments were required.

**Table 1:** Definitions of supports.

Key concept:	Definition Includes:
**Assistive Technology (AT) Devices **	Full Use of ISO 9999 Assistive Products for Persons with Disability * Products and Technology Chapter 1 ** Independent Living Centres Australia Product Database Structure (personal communication)
**Environment**	iv Furnishings and adaptations to homes and other premises & Assistive products for environmental improvement, tools and machines * (pp. 40, 55) v Natural Environment and Human Made Changes to Environment Chapter 2 ** (p. 182) vi Independent Living Centres Australia Product Database Structure (personal communication)
**Personal Care**	vii Support and relationships Chapter 3; Attitudes Chapter 4; Services, systems and policies Chapter 5 (pp.187, 191, 192)

* ISO Assistive products for persons with disability—Classification and terminology; ISO: 2007; ** World Health Organisation. International Classification of Functioning, Disability and Health. World Health Organisation: Geneva, Switzerland, 2001.

#### 2.2.4. Data Analysis

Data analysis was performed by the first author, triangulated with her PhD supervisor, and member-checked with the advisory panel. The initial analysis identified which supports were used to enable activities and participation. Participants were also asked what additional or alternate supports or accommodations would enable them to achieve further, self-determined outcomes. Statistical advice suggests the heterogeneous sample did not lend itself to subgroup analyses by participants’ specific demographic features (age, gender, living situation). Table 1 shows the matrix used to categorize the range of supports during initial analysis, when coding each data unit in the excel spreadsheet.

Some of these classification systems reflect differing degrees of detail, but when used together, they presented a finely grained set of definitions that exhausted the data categorization. The Equipment Study provided empirical evidence for the working hypothesis of the “AT solution”:

An AT solution is an individually tailored combination of hard (actual devices) and soft (assessment, trial and other human factors) assistive technologies, environmental interventions and paid and/ or unpaid care.

*[29]*

Participants also described a diversity of enabling elements relating to, but moving beyond, environmental modifications in the community. It is likely the experience of environment is also influenced by personal factors such as gender, age, and disability type as well as income, compensable status, and employment. The sample size and study structure did not enable this level of analysis, but this is certainly a topic for further inquiries. Spreadsheet data were qualitatively analyzed against the concepts of the natural and built (tangible) environment, and other (intangible) dimensions of participants’ home and community environments. Hence a discrete category “inclusive community environs” was created as a subset of the environment component of an AT solution, expressing the combined impacts of built and intangible aspects of environments capable, in transaction with individuals, of creating or eliminating the experience of disablement. 

## 3. Results and Discussion 

### 3.1. Health-Related Quality of Life

AQoL scores are reported between 0 (death) and 1 (excellent health). Scores approaching 1 are deemed high, representing high life quality and excellent health; scores below zero are categorized as “states worse than death” [28]. The survey population achieved a mean score of 0.32 in relation to the population’s current health-related quality of life (Table 2). This contrasts with an Australian population mean of 0.80 [28]. Survey respondents reported mean AQoL scores lower than half those of the Australian reference population for both genders. They demonstrated a large range in AQoL scores, from −0.1726 to 0.885. These results demonstrate an overall struggle for the population of people with disability to achieve the quality of life of the general Australian population, and a lower level of functioning across the six domains of the AQoL than is the Australian norm.

### 3.2. The Range of Supports (Elements of AT Solutions) Needed to Participate

Overall, The Equipment Study demonstrated that people with disability utilize an average of 13 supports to live their lives, usually drawn from multiple sources and funders. As shown in Table 2, two thirds (66%) used a combination of AT devices, environmental interventions and personal care: that is, an AT solution. A further 16% of participants used combinations of AT devices and personal care; 15% used AT devices and environmental interventions together; and 2% used AT devices alone. The majority of participants (74%) identified unmet needs.

**Table 2:** Number of supports used or required by participants (N-100).

Supports or Elements of AT Solutions Currently in Use or Required	Currently in Use (*i.e.*, Met Need)		Currently Required (*i.e.*, Unmet Need)	
	No. of Elements/Items	% of Respondents	No. of Elements/Items	% of Respondents
Assistive Technology Devices	768	97%	156	70%
Environment:				
Home Modifications	365	43%	70	46%
Community environs	24	20%	138	52%
Personal Care	176	81%	33	24%
**Total**	**1333**		**397**	

### 3.3. Inclusive Community Environs as a Subset of Environment

Inclusive or accessible community environs emerged as a discrete category and theme from the data. Participants classified a wide range of aspects of environment as either facilitators or barriers. Table 3 presents examples of these, classified according to the ICF chapters dealing with environment.

**Table 3:** Inclusive community environs examples by ICF chapter.

**Chapter 1 Products and Technology**	
Online environment as a barrier	Online environment as facilitator
I want to provide training and education to people. I would love a notebook computer so I could take it with me when I went out … take information out with me, not be stuck in my room. [S110]	My computer is my window to the world. I use it to keep in touch, to do research, pay bills, order groceries and buy from eBay. [S42] I use my lightwriter for communication. I go shopping with my mother weekly using my walker, I use telephone banking. [S55] As I mainly use public transport I spend time planning on internet the route and means of transport (timetables) and how they connect. [S47]
**Chapter 2 Natural environment and human-made changes to environment (WHO 2001)**	
Physical environment as barrier	Physical environment as facilitator
Access in my neighbourhood is very poor and I’m not confident at all getting around. Some places are too steep and some places don’t have footpaths… neighbourhood access for wheelchairs would make things a lot easier. [S88]	I can shop at the green grocer, baker, and small food shop on my own, the people know me and the shops are accessible. [S26]
Belong to the local Interfaith network. My church is in Melbourne and no trams there yet—got the stops but no accessible trams on that line! [S15]	Physical access can be provided by my portable ramp. [S9]
**Chapter 3 Support and Relationships and Ch. 4 Attitudes**	
Attitude as a barrier	Attitude as facilitator
The station staff could be more willing and ready to help, if they see the person with the disability is having trouble getting a ticket out of the ticketing machine! [S44] An impossible change—people’s attitudes, just because I am in a chair I am not stupid!!!! [S26]	I rely on friends to drag me up steps *etc.* [S25] My local [Member of Parliament] has a step up to his office, landlord will not allow a permanent ramp. His staff are very good, if they hear me they bring out a portable ramp, however the council will not allow it to be left set up while I am visiting!!! [S26]
**Chapter 5 Services, systems and policies (WHO 2001)**	
Systems as a barrier	Systems as a facilitator
[I would like] no stairs, plenty of places to sit and rest, public transport stops closer together, wider and more accessible toilets (not just disabled ones), disabled toilets not being “key available on request”. [I would like] a cut in path in my nature strip near my front door as the nearest cut in the gutter is up the road which when getting a taxi I get rather wet, council will not let me do it even though I was willing to pay [S81]	If I fly to a destination the airlines are very good they arrange a wheelchair and a person to assist with all my needs. [S55] I am a Celebrant and my attendant carer provides access to every venue and does all of the physical work to allow me to perform and take part in educational and celebrancy life [S106]

Barriers in community environs were reported by over half the participants (52%). Of these, the reported need was greatest for universal design of and physical access to outdoor environs and buildings, and accessible public transport and public space. Examples and incidence of these are presented in Table 4.

**Table 4:** Descriptions, examples and incidence of unmet need for inclusive community environs.

Community Environs	Examples of Unmet Need	Incidence of Unmet Need
Public buildings	Universal design of buildings including: stepless entry; easy doors; presence of accessible toilets; appropriate height reception/sales desks at shops and other venues; seating; accessible swimming pools/gym	49
Public transport	More low floor buses, accessible tram stops, large print and talking timetables	33
Public space	Footpaths, kerb access, tactile street signage; street crossings; accessible parking (presence of disabled parking spots; proximity to destination)	36
Public information and support	Accessible information on websites including information as to whether access is possible at venues written in accessible formats; helpful and trained staff	14
Income support and supplements	Increase in pension and allowances; savings; recourse to top up funds to purchase supports	6
**TOTAL**		**138**

The Equipment Study data supports the concept of a multifaceted environment, comprising built and non-physical elements of places capable of creating, or eliminating, the experience of disablement. Analysis of the emergent theme “inclusive community environs” provides an operational definition of the built and human elements of public places that environment represents to this cohort of people with disability. The surface and content validity of the concept were confirmed through triangulation with participants and with a steering group including people with disability. 

### 3.4. Multiple Dimensions of the Environment

Responses show the link between impairment and environmental barriers in creating disability. Participants frequently reported a mix of both barriers and facilitators, providing concurrent experiences of inclusion and disablement:

Being able to do my own shopping is a great pleasure and an independence event [but] when a shop is only partly accessible and the specials are in an area where there is no accessibility then I have to go home without a moment of equality.

*[S35]*

The following comments from participants are typical of issues they reported facing in public urban spaces and of the unmet need for modifications:

Easy access to buildings would save huge amounts of time and stress. The good footpaths would mean I did not get tired so quickly and therefore could be out in the community doing what I wanted for longer periods of time. Good public transport is obvious.

*[S26]*

Counters at a usable/accessible/reachable level in government and private offices and businesses. Microphone podium accessibility. Lifts that work and don’t stop a few centimetres off or below the floor level. Better emergency escape plans regulated.

*[S35]*

[I need] street changes—I use a chin-controlled chair and when I try to move the chair along street paths and cross the road, poorly constructed bumpy and steep crossovers are extremely difficult to navigate with my chin. When paths are not flat and smooth, my head moves too much for my chin to remain on the chin control, it makes it nearly impossible for me to get out in most areas locally like to the park or shop. Roads are more smooth than paths. The use of blue stones for crossovers is appalling for wheelchair users. [I need] better access into some buildings, venues and shops that haven’t provided access for the disabled in wheelchairs.

*[S89]*

Social and attitudinal aspects of environments were also described as integral to the outcomes of participation and inclusion and the experience of disablement. One young adult with vision impairment who uses a wheelie walker to mobilize described her life as follows:

I am extremely competent with public transport—however I don’t go to large events such as the football and concerts on my own—it is too easy for me to be knocked over in large crowds.

*[S38]*

In this instance, public transport is used with support from public transport staff to access a range of activities and venues. Participating independently in major events, however, is avoided for safety reasons. Other participants commented on more global experiences:

Misinformation in relation to what some venues/locations call “accessible” e.g., “It only has one small step”, “there is just a step into the shower”, “Parking is close by” etc.

*[S30]*

This result builds on the evidence base [30,31] for ecological models of health, and illustrate a transactive relationship between individuals performing activities and the environments in which this occurs, as illustrated in occupational therapy theory [17].

### 3.5. Validation of Concepts to Capture Disablement Experiences Related to the Environment 

As the data demonstrate, the impact of environments upon an individual and their occupations is at once individualized, nuanced and subjective. Environmental factors which present a barrier for some, are a facilitator for others. The most potent source of measurement is the person themselves, and the centrality of the person’s experience requires inclusive methodologies and meaningful taxonomies. Both objective and subjective data can be captured within the ICF’s chapters of environmental factors, though they lack the specificity of occupational therapy’s ecological theories [32]. 

Given the diversity of environmental barriers and facilitators that exist for people, professionals must apply a wide conceptual lens and specificity when naming the impact of environment upon health and wellbeing. This includes definitions of environment that extend beyond the built environment to social, cultural, and institutional dimensions. In addition to physical access, participants described inclusive elements such as buildings with operable doors and lifts, accessible counters, and educated and friendly staff. Attitudinal aspects of the environment were identified as enormously important to the experience of inclusion by participants. The ICF, with its language of environmental barriers and facilitators, provides a taxonomy to capture experiences across both tangible and intangible aspects of environments [33]. 

Another valuable concept encompassing the subjective nature of inclusion is that of “usability”, which enables an individual evaluation incorporating psychosocial factors and perceptions of how well an environment enables participation and inclusion [24]. This may be helpful in progressing beyond regulation for accessibility, which is often perceived as prescriptive or homogenous. As Priestley *et al.* (2009) describe,

A holistic approach to the “usability” of physical environments needs to be applied … which goes beyond technical requirements for physical access. Confidence, security, information and forms of social interaction and assistance are all relevant to the usability of physical space.

*[34] (p. 78)*

Findings from The Equipment Study highlight the need for collective, rather than individual responsibility for creating inclusive environments. Participants identified barriers that limit their participation and “cost” them their inclusion; a cost borne individually. Recent work outlines a “reversed” position of responsibility with respect to access and inclusive communities, that focuses on the cost of non-social [35] and non-inclusive environments [24,36]. Termed the “economics of inclusiveness”, costs are calculated over all community members (parents with prams, elders with shopping trolleys *etc.*) who may benefit from interventions such as kerb cuts and accessible transport [37,38]. Such a shift in approach and accounting would mean, for example, that architects conceive access not as a constraint upon design, but as a “major perceptual orientation to humanity” [39] (p. 112). 

### 3.6. Implications for Roles and Responsibilities of Individuals, Professionals, and Society 

On an individual level, The Equipment Study enabled people with disability to demonstrate the disabling impact of a mismatch between environments and capacities: data which mirrors global experiences [40]. Individual participants identified many possibilities for removing existing environmental barriers, and described potential outcomes should this occur. Such changes directly relate to improved inclusion experiences, as can be seen in the following example:

I stayed in Seattle where it is more physically accessible. I realized I felt different and the difference was that I felt more like I used to feel before my accident when I lived in an environment that was built to include rather than exclude me.

*[S25]*

The finding that multiple levels of environmental change are required, challenges the role of professionals. Perhaps due to the dominance of biomedical discourses, the locus of intervention within environments for occupational therapists for example has predominantly addressed local adaptation of the built environment related to an individual and their impairment. 

Environmental interventions are part of the occupational therapy scope of practice [41], and there is strong congruence between occupational therapy theory and disability theory regarding issues of person and environment fit, evidenced in occupational therapy’s transactive person-environment models, in which people perform tasks or activities that engage time and energy, known as occupations, within environments [42,43]. Indeed, this has been the basis of key occupational therapy interventions, whereby environmental design (such as continuous paths of travel or workstation design), provision of supports such as assistive technology (AT) devices (mobility aids, alternative and augmentative communication aids) or accommodation through adaptations (widening doors, installing grab bars) are introduced to improve access generally and minimize the capability gap [44] between the person and the task. 

Some excellent texts describe a range of strategies to improve person–occupation–environment fit, but acknowledge the difficulty enacting systemic changes that would benefit communities and societies [45,46]. Beyond remediating issues of individual ‘fit’ between one individual and one environment, this transactive understanding of human functioning and health in context, along with human rights advocacy by people with disability, has informed society’s understanding of the nature of disability and highlighted discrimination and exclusion that occurs due to barriers in public buildings and services. This means, rather than being perceived as outside the scope of professionals’ practice, advocacy for systemic change is necessary to bridge the capability gaps identified by people with disability [47]. Restricting environmental interventions to local adaptations risks a failure to respect human rights, and misses the opportunity to facilitate widespread inclusion across communities. 

Systemic change in community environs is usually enacted by local and regional government authorities or individual businesses. This is often in response to complaints under disability discrimination legislation, or as part of regulatory procedures in building and planning. Examples include visual and tactile surfacing of roads and paths; retro-fitting entrance ramps, or providing “disability awareness” training. From a human rights perspective, the state has a key role in supporting inclusion through its tools of public administration; usually laws and regulations [48]. Pursuing regulation is one obvious route to change, however some authors question the use of regulation as the primary avenue of change, as it may position inclusion as a compliance problem [49]. Tackling barriers through legal action may result in ‘isolated legal victories [which] will provide little correction to what are otherwise entrenched manifestations of societal exclusion’ [50] (p. 273). Non-regulatory policy instruments that can change cultural attitudes, as well as physical infrastructure, are required [51].

On a societal level, the disability rights movement has clearly articulated the social and political nature of disability, diluting the dominance of biomedical discourses and prompting legal recognition of a more holistic notion of health and wellbeing [15]. Societal responsibilities for health and inclusion are recognized in international legislation drawn from a human rights perspective. The complexity of translating disability rights into improved outcomes, even with political support and regulatory incentives, is acknowledged in other studies [52]. Findings in this paper illustrate the imperative for professionals working with humans and with the environments and contexts in which humans live to identify and address mismatches that result in disablement. Professionals should advocate for and enact systemic, as well as local environmental change, and be pro-active to ensure that benefits are shared across communities.

## 4. Conclusions 

The environment provides context for human endeavor and is a critical variable to be considered in relation to inclusion of people with disability. This article presented an in-depth analysis of a cohort of individuals living with disability which empirically demonstrates the impact of broadly defined environmental facilitators and barriers upon inclusion and wellbeing.

The results support the theoretical premise identified in the literature whereby disablement results from a mismatch between individuals’ capabilities (based upon impairment effects and personal factors), the particular environments in which they live, and the occupations they enact. Further, results indicate that when environments fully accommodate an impairment, it may cease to be experienced as disability. Examining the role of environment in relation to impairment is essential to ascertain the extent of disablement and the ways to mediate this.

Any one individual has limited agency within societal structures. People with disability experience higher costs of disability, a thinner margin of health, and vulnerability to significant capability gaps in non-inclusive environments [40]. Despite this, systemic advocacy by people with disability has resulted in the CRPD [22] and anti-discrimination legislation. Systemic environmental changes necessitate collective responsibility for the creation of inclusive environments, rather than reliance on individual agency or governmental regulation. Professionals hold moral and ethical responsibilities in relation to inclusive environments and are encouraged to perceive and co-ordinate action on environmental barriers. This entails focusing on the standpoint of the person with disability and addressing the multiple tangible and intangible aspects of environment which influence their outcomes, at all available opportunities. Competent and ethical professionals, working with people with disability and across disciplines, are in a strong position to enact change that enhances wellbeing and inclusion.
